# IMPLEMENTING A GERIATRIC FRACTURE PROGRAM IN A PLURALISTIC ENVIRONMENT REDUCES LENGTH OF STAY AND TIME TO SURGERY

**DOI:** 10.1093/geroni/igz038.2710

**Published:** 2019-11-08

**Authors:** Sonja L Rosen, Kathy Breda, Carol Lin, Jeanne Black, Jae Lee, Aaron Chiang, Mark Vrahas, Brad Rosen

**Affiliations:** Cedars-Sinai Medical Center, Los Angeles, California, United States

## Abstract

Geriatric-orthopaedic co-management models have been demonstrated to improve patient outcomes, but are typically implemented in closed, non-pluralistic medial systems. The Cedars-Sinai Geriatric Fracture Program (GFP) was developed through collaboration amongst a multi-disciplinary group. Cedars-Sinai is an academic medical center with a pluralistic medical staff that includes faculty, several hospitalist groups, and private practitioners. The GFP was introduced in July 2018 as a quality improvement pilot to provide standardized treatment for geriatric fracture patients. We hypothesized GFP enrollment would reduce time to surgery (TTS) and length of stay (LOS). Geriatric fracture patients were prospectively enrolled from July -December 2018. The Wilcoxon Rank- Sum test was used to compare TTS and LOS between the two patient groups. A p < 0.05 was considered significant. 190 operative fractures in patients over 65 years-old were prospectively followed.56 (30%) were enrolled in the GFP, 54 (28%) were admitted to other hospitalist groups (OH), and 80 (42%) were managed by their primary care physician (PCP). There were no demographic differences between groups. Patients enrolled in the GFP had a significantly shorter LOS compared to the OH and PCP groups (4 days v 5 days v 5 days, p = 0.039) as well as a significantly shorter TTS (19.7hrs v 22.4 hrs vs 23.3 hrs, p = 0.037). Our data shows that a multi-disciplinary geriatric fracture program can be successfully implemented in a complex pluralistic environment resulting in improved patient metrics. Adherence to evidence-based protocols and close multidisciplinary teamwork are critical to program success.

